# Rambling

**Published:** 1883-10

**Authors:** J. B. Chandler


### RAMBLING.

J. B. CHANDLER.

Whilst lingering upon the shores of Lake Ontario, with mind attuned only to plans for inveigling the bass and wily pickerel from their haunts, comes the announcement that it is BISTOURY time, and our usual contribution is needed.

We have not escaped the vicissitudes of life, indeed, we imagine that our scars have been as deep as usually fall to the lot of man, but we have to be thankful now, when we are approaching the period of life, as allotted by the Scripture, that our journey down the hill is lightened by an absence of care, and the possession of means and tastes to enjoy, in a quiet way, pleasures and sports suitable to age.

Early in the Spring we gathered the arbutus, and the violets budding and blooming beside the trout streams of Eastern Pennsylvania. Later on, with guide and canoe, we sought the speckled beauties amidst the lakes and streams of the Adirondacks, noting the evergreens pushing out fresh shoots amidst the olive foliage of the past year, and the graceful tamaracks clothing themselves in Summer foliage. When the black-flies assumed their rule and drove us from the North Woods, we gathered azalias and daisies on the hills around Elmira. Then a few weeks among the bass and water-lilies at Sodus. Thence we were summoned by telegraph to Philadelphia, to the death-bed of one of our nearest and dearest friends. One of the few whose friendship bore the test of trial. The chum with whom for years we have waded the trout streams of the Alleghanies; shoulder to shoulder, making right and left casts, with no rivalry as to results, except that the delivery of each should be worthy of the other, and that the counterfeit should imitate in its motions the natural ephemera so closely as to deceive the wily trout. He has gone; a noble-hearted man, true friend, and good fisherman. We were with him to the end; assisted in the last rites, and offered what we could of consolation and advice to the near and dear ones he left behind. When our fishing days are over, and we are called away, may our record be as good as his.

Leaving these sad and painful scenes, again we find ourselves seeking a toning of grief at one of our favorite haunts. Time and action will bring, not forgetfulness, but at least a peaceful acceptance of the inevitable.

Now, kind reader, what can you expect in the way of thinking, solid matter from a writer just so situated? Of course in travel to and fro we have overheard remarks and discussions on many questions, we have sat for several hours in a car listening to the arguments of a delegate to the Free Thinkers Convention upon the subject of a "previous," "present," and "future" existence. We have listened to advanced theories upon Blood Corpuscles, indeed, have found time and opportunity to examine and note the difference between the corpuscles of the Amphiuma, the toad, the dog and those of man. We have been eye-witness to various scenes of life, which would make excellent foundations for stories, or a novel. Those who move about freely, encounter human nature in many phases, and let it be noticed that they are very unfortunate if they do not find much in it that appeals to the finer feelings of the heart.

Autobiography, however free from exciting passages it may be, nevertheless is not entirely devoid of interest. Perhaps this little bit of *Ego* will be forgiven in the lesson it may convey, that a man may pass the greater part of his life in the struggles and cares of business, and yet retain feelings and tastes which will give him something else to do, in idle days of age, besides nursing rheumatism and dying of *ennui*. Stick to your business during business hours, but keep alive the tastes and faculties for enjoyment, so that when the active duties of life are discharged, and the days draw near the end, you shall still find some pleasures in them.

